# Hybrid regulatory T cells: camouflaged architects of tumor immunity

**DOI:** 10.3389/fimmu.2025.1658576

**Published:** 2025-09-17

**Authors:** Parviz Azimnasab-Sorkhabi, Maryam Soltani-asl, Jose Roberto Kfoury Junior, Ephraim A. Ansa-Addo

**Affiliations:** ^1^ Division of Medical Oncology, Department of Internal Medicine, The Ohio State University College of Medicine, Columbus, OH, United States; ^2^ Pelotonia Institute for Immuno-Oncology, The Ohio State University Comprehensive Cancer Center Arthur G. James Cancer Hospital and Richard J. Solove Research Institute, Columbus, OH, United States; ^3^ Department of Surgery, School of Veterinary Medicine and Animal Sciences, University of Sao Paulo, Sao Paulo, Brazil

**Keywords:** regulatory T cells, hybrid tregs, T-bet⁺ hTregs, GATA3, Foxp3, tumor microenvironment, immunotherapy

## Abstract

Distinct from conventional Foxp3^+^ regulatory T cells (Tregs), T-bet^+^ Tregs represent a stable subset of immunosuppressive T cells characterized by co-expression of the transcription factors (TFs) Foxp3 and T-bet. Given that Tregs were also reported to co-express Foxp3 together with effector T cell TFs such as GATA3, or RORγt, we propose the term hybrid Tregs (hTregs) to distinguish between these Tregs that co-express Foxp3 together with effector T cell TFs from conventional Foxp3^+^ Tregs. Therefore, this review will focus on hTreg cells, a specific subset of CD4^+^ T cells, and discuss the different types of hTregs with particular emphasis on T-bet^+^ hTregs. T-bet^+^ hTregs exhibit unique features including IFN-γ production, high expression of immune checkpoints (PD-1, CTLA-4, GITR, OX40, TIGIT), and chemokine receptors (CXCR3, CCR5). Through secretion of IL-10, TGF-β and IFN-γ, T-bet^+^ hTregs modulate both innate and adaptive immune responses within the tumor microenvironment (TME). Their high expression of CD73 contributes to adenosine-mediated immunosuppression, while CXCR3 and CCR5 facilitate their recruitment to inflammatory sites. T-bet^+^ hTregs were reported to accumulate in multiple human cancers, including lung, ovarian, and colorectal carcinomas. Despite these advancements, the function of hTregs in diseases such as cancer remains poorly understood, and requires further investigations. For instance, some studies suggest T-bet+ hTregs to be anti-inflammatory due to their production of IL-10, TGF-β, and superior suppressive capacity compared to conventional Tregs. Yet, other studies have reported that T-bet^+^ hTregs exhibit enhanced proinflammatory functions in colitis and other pathologies. We will then highlight current known mechanisms that promote the differentiation and functions of T-bet^+^ hTregs in cancer. Lastly, we will discuss the advancements and opportunities for therapeutic targeting of T-bet+ hTregs in cancer immunotherapy.

## 1 Introduction

The tumor microenvironment (TME) comprises of cancerous cells, and a diverse array and interaction of non-cancerous cells, including stromal and immune cells (1). Within such an ecosystem, the diversity of cell-cell interactions, cellular synapses, and secretome within the TME define the fate of tumor progression, metastasis rates, and treatment outcomes in patients (2–4). Tumor infiltrated T cells (TILs) exhibit substantial heterogeneity in their phenotypes, functional states, and spatial distributions within tumors (5, 6). Regulatory T cells (Tregs) that express the transcription factor Foxp3 are among the most abundant T cell subsets within the TME (7). Similar to conventional T cells, Tregs respond to T cell antigen receptor (TCR) stimulation by transitioning from a resting state to a more suppressive effector Treg (eTreg) phenotype (8). Although, the immunosuppressive capacity of Tregs play an indispensable role in maintaining tolerance and immune homeostasis, it suppresses antitumor immune responses in the TME (7, 9). Currently, one of the challenges in the Treg field is successfully manipulating Tregs in cancer without triggering autoimmunity. However, Tregs should not be considered as a homogeneous population. During the last decade, different hybrid subsets of Tregs such as Th1-like Tregs and Th17-like Tregs have been discovered in various human and mouse diseases (10). Nevertheless, the terms “Th1-like” or “Th17-like” Tregs, which have been widely used do not properly describe these unique cells. For instance, in cancer, T-bet^+^ Tregs exhibit characteristics that more closely resemble conventional Tregs than Th1 cells. Likewise, RORγt^+^ Tregs and GATA3^+^ Tregs display properties that are similar to conventional Tregs than their helper T cell counterpart. Simply, these cells are not equivalent to the helper T cells and only partially share certain characteristics. We propose the term ‘hybrid Tregs’ to reflect their expression of two T cell lineage master transcription factors that regulate T cell fate and differentiation. Henceforth, we will use the term hybrid Tregs (hTregs) to refer to these subsets of regulatory T cells. Notably, hybrid subsets of Tregs are functionally different, compared to conventional Foxp3^+^ Tregs. For example, while conventional Tregs broadly suppress diverse immune responses, T-bet^+^ hTregs specifically target and suppress Th1-type immune responses and associated inflammation.

T-bet^+^ hTregs are defined by the co-expression of the Th1 cell transcription factor T-box transcription factor TBX21 (T-bet) and the Treg signature transcription factor Foxp3 (11). A significant accumulation of T-bet^+^ hTregs is reported in several tumors including oropharyngeal squamous cell carcinoma and lung carcinoma (11, 12). T-bet^+^ hTregs exhibit high resistance to oxidative stress, which may contribute to their accumulation in tumor tissues (13). In addition, T-bet^+^ hTregs primarily execute their immunosuppressive characteristics by dampening type 1 immune responses including the activation of Th1 cells and cytotoxic CD8^+^ T cells which are essential for antitumor immunity and defense against intracellular pathogens (14). Despite their immunosuppressive functions and abundance within the TME, the precise contribution of T-bet^+^ hTregs to tumor progression remains poorly understood. Given that they share features with both Tregs and Th1 cells, T-bet+ hTregs were suggested to influence the tumor immune landscape by suppressing antitumor immunity, yet their exact role in promoting or inhibiting tumor growth, metastasis, and response to immunotherapy remains less understood. In this review, we aim to shed light on the possible functional mechanisms of T-bet^+^ hTregs by exploring their resemblance to conventional Tregs.

## 2 Plasticity of Tregs and hybrid Tregs within tumors

Given the central role of conventional Tregs in regulating and maintaining immune homeostasis, they require steady and robust suppressor functions. Nonetheless, Tregs have been shown to exhibit substantial plasticity which enables them to effectively suppress a wide range of immune responses. Although this flexibility is driven by intrinsic molecular signaling pathways, such as the PI3K/AKT pathway, the activation of these pathways is also influenced by the surrounding environment of the Tregs, including various cytokines and metabolic factors (15, 16). This adaptability gives rise to specialized subsets of Tregs called hybrid Tregs which further refine immune regulation. Hybrid Tregs co-express lineage-defining transcription factors typically associated with other T helper (Th) subsets, such as T-bet (Th1), GATA3 (Th2), RORγt (Th17), and Bcl6 (Tfh) (16). These hybrid populations are thought to be crucial for suppressing their corresponding effector T cell counterparts. For instance, RORγt^+^ hTregs play a role in regulating Th17 responses in the gut (17).

### 2.1 T-bet^+^ hTregs

The frequency of T-bet^+^ hTregs among CD4^+^ T cells in the peripheral blood of healthy humans has been reported to be less than 2% (~6 cells/µL). In contrast, their frequency among memory Tregs has been reported to be approximately 40% in rheumatoid arthritis (18). Under specific inflammatory conditions T-bet^+^ hTregs differentiate from Tregs and their presence in different disease including Type 1 diabetes, Rheumatoid arthritis, and cancer have been reported (18, 19). Like conventional Tregs, T-bet^+^ hTregs also express Foxp3 and Helios, but in addition, they upregulate T-bet and CXCR3 (20). In settings with elevated cytokines including IFN-γ, interleukin-27 (IL-27) and IL-12, conventional Tregs sense and upregulate T-bet, as well as secrete IFN-γ and TGF-β, and exhibit other Th1-related markers such as CXCR3 and CCR5 (13, 19, 21–23). **Table 1** provides a detailed comparison of key characteristics among human T-bet^+^ hTregs, GATA3^+^ hTregs, and RORγt^+^ hTregs, highlighting their unique profiles, including transcriptional profiles and cytokine production (24, 25).

**Table 1:** Comparison of key characteristics among T-bet^+^, GATA3^+^, and RORγt^+^ hTregs.

Cell type Characteristic	T-bet^+^ hTregs	GATA3^+^ hTregs	RORγt^+^ hTregs
Key transcription factors	FoxP3, T-bet	FoxP3, GATA3	FoxP3, RORγt
Foxp3 expression (MFI)	> 1500	> 1000	~ 1500
CD25 expression (MFI)	< 6000	< 6000	~ 7000
PD-1 expression (MFI)	~ 2000	> 4000	< 4000
CTLA-4 expression (MFI)	~ 500	< 1000	> 500
TIGIT expression (MFI)	~ 3000	> 4000	>3000
GARP expression (RNA-seq)	Low expression	Low expression	High expression
Secreted cytokines	IFN-γ, TNF-α	IL-2, IL-4, IL-5, IL-13, IL-21	IL-17A, IL-17F
Upregulated Th-lineage genes	IL7, IL15, TBX21, IRF1, CXCR3, IFNg	GATA3, FOSL1, IRF4, IL1A, IL4, IL5, IL9, IL13, IL21, IL24, IL2, CEBPB, IRF8, RUNX3, IL6, NFATC1	RORA, RORC, RUNX1, CCR6, IL17A, IL17C, IL7, IL15
Upregulated chemokine receptors	CCR2, CCR5, CXR6, CXCR3	CXCR5, CXCR4	CCR2, CCR5, CXR6, CCR6, CCR9
Upregulated chemokine	Not Available	CCL24, CXCL8, CXCL16, CCL3, CCL17	CXCL13

The presence of T-bet^+^ hTregs in the TME and lymph nodes of cancer bearing patients suggests that the TME is not the sole site where these cells can differentiate. Instead, T-bet^+^ hTregs are likely recruited and retained in the TME, contributing to their high abundance in this environment (14). Tumors can attract and retain CXCR3-expressing cells through the secretion of specific chemokines, primarily C-X-C motif chemokine ligand (CXCL) 9, CXCL10, and CXCL11, which are ligands for the CXCR3 receptor. These chemokines create a gradient that guides CXCR3-expressing immune cells, such as activated CD8^+^ T cells and natural killer (NK) cells, toward the tumor site (26, 27). Also, CXCR3 plays a crucial role in stabilizing intravascular adhesion of T cells, facilitating their extravasation into the tumor tissue (28). Understanding the mechanisms governing the recruitment and retention of CXCR3-expressing cells including T-bet^+^ hTregs within the TME is crucial for developing therapeutic strategies aimed at modulating immune cell infiltration to improve antitumor immunity.

### 2.2 GATA3^+^ hTregs

GATA3^+^ hTregs are known to suppress Th2 cell responses (29). The function of Th2 cells in tumor response is complex and multifaceted, with evidence suggesting context-dependent antitumor and pro-tumor effects. Th2 cell were suggested to impair the growth of colon and pancreatic tumors by secreting cytokines, including IL-4, IL-5, and IL-13, which can recruit and activate eosinophils, and other cytotoxic cells into the TME (30, 31). In breast cancer, Th2 cells were shown to induce terminal differentiation of cancer cells, effectively suppressing their malignant potential (32). Of note, Th2 cell responses can also promote tumor growth and metastasis (31). Tregs require IL-4 to differentiate into GATA3^+^ hTregs (33). GATA3^+^ hTregs are abundant in melanoma and colorectal cancers. While T-bet^+^ hTregs and RORγt^+^ hTregs are prevalent hybrid Tregs in colon, GATA3^+^ hTregs were found preferentially in tissues compared to the circulation, even in the skin and colon. Although lower expression of Foxp3 is observed in GATA3^+^ hTregs compared to T-bet^+^ hTregs and RORγt^+^ hTregs, these cells are characterized by high levels of IL-2, IL-4, IL-5, and IL-13 cytokines and increased chemotaxis toward CCL17/22 compared to other hybrid Tregs.

GATA3^+^ hTregs demonstrate superior survival and enhanced proliferative capacity driven by the autocrine IL-2/STAT5 signaling pathway. Additionally, these cells exhibit reduced suppression of Th2-like effector T cells relative to other Treg subsets, likely due to their elevated expression of TIGIT, the only key protein differentially expressed upon activation. Due to enhanced survival, greater migratory potential, and specific suppression of effector T cells, it is suggested that GATA3^+^ hTregs might promote a tumor-supportive environment (24). GATA3^+^ hTregs exhibit unique characteristics that may contribute to a tumor-supportive environment, emphasizing the need for further investigation into their role in cancer to uncover potential therapeutic opportunities.

### 2.3 RORγt^+^ hTregs

Tregs require IL-6, IL-21, and IL-23 to differentiate into RORγt^+^ hTregs with capacity to produce IL-17A (33–35). RORγt^+^ hTregs that secrete IL-17 and express CCR6^+^ exhibited the highest levels of RORγt, indicating a close resemblance to Th17 cells (36). They are known to suppress Th17 cell responses (37, 38). Th17 cells, particularly through the secretion of IL-17A, can promote tumor angiogenesis (39). Th17 cells contribute to tumor progression as IL-17 triggers IL-6 production, which activates oncogenic signaling pathways, including STAT3. STAT3, in turn, promotes tumor growth by regulating genes involved in angiogenesis (40). It is shown that RORγt^+^ hTregs are the dominant population in the skin, while the colon is enriched with T-bet^+^ hTregs and RORγt^+^ hTregs (24). The equilibrium between Th17 and Tregs is governed by the glycolytic pathway driven by mTOR signaling (41). Overall, there is a lack of comprehensive studies investigating the role of RORγt^+^ hTregs and their interaction with Th17 cells in tumor immunity. Understanding this dynamic could provide valuable insights into the balance between pro-tumor and antitumor responses with potential clinical applications for targeting these cell populations in cancer therapies.

### 2.4 T follicular regulatory (Bcl-6^+^ hTregs) cells

CD4^+^ T follicular helper (Tfh) cells play a crucial role in supporting B cell functions and are commonly located within tertiary lymphoid structures in tumors. In malignancies derived from Tfh cells or B cells, an elevated frequency of Tfh cells is often linked to poor outcomes. In contrast, in solid organ tumors of non-lymphocytic origin, higher Tfh cell levels are frequently associated with improved prognoses, underscoring their dual and context-dependent roles in cancer (42). In Tfh cells, STAT5 and Bcl-6^+^ have been reported to bind to shared DNA sequences (43). Bcl-6^+^ hTregs produce IL-10 and TGF-β, and play a complex and dual roles in tumor immunity (44). Anti-PD-1 and anti-CTLA-4 monotherapy significantly increased the frequency of Bcl-6^+^ hTregs within tumors (45). Given Bcl-6^+^ hTregs’ complex and dual role in tumor immunity, further investigation into Bcl-6^+^ hTregs is crucial to understanding their impact on antibody responses and potential therapeutic strategies in cancer treatment.

## 3 T-bet^+^ hTregs in tumor immune regulation

The role of T-bet^+^ hTregs in cancer is multifaceted and highly context-dependent, exhibiting both pro-tumor and antitumor functions. This duality highlights their remarkable plasticity and ability to adapt to the TME while preserving their regulatory phenotype. It is suggested that T-bet^+^ hTregs exhibit more rebuts inhibitory capacity in comparison to other hybrid Tregs (18). The inhibitory function of T-bet^+^ hTregs might rely on different mechanisms, including:

### 3.1 Cytokine production

**Figure 1:**
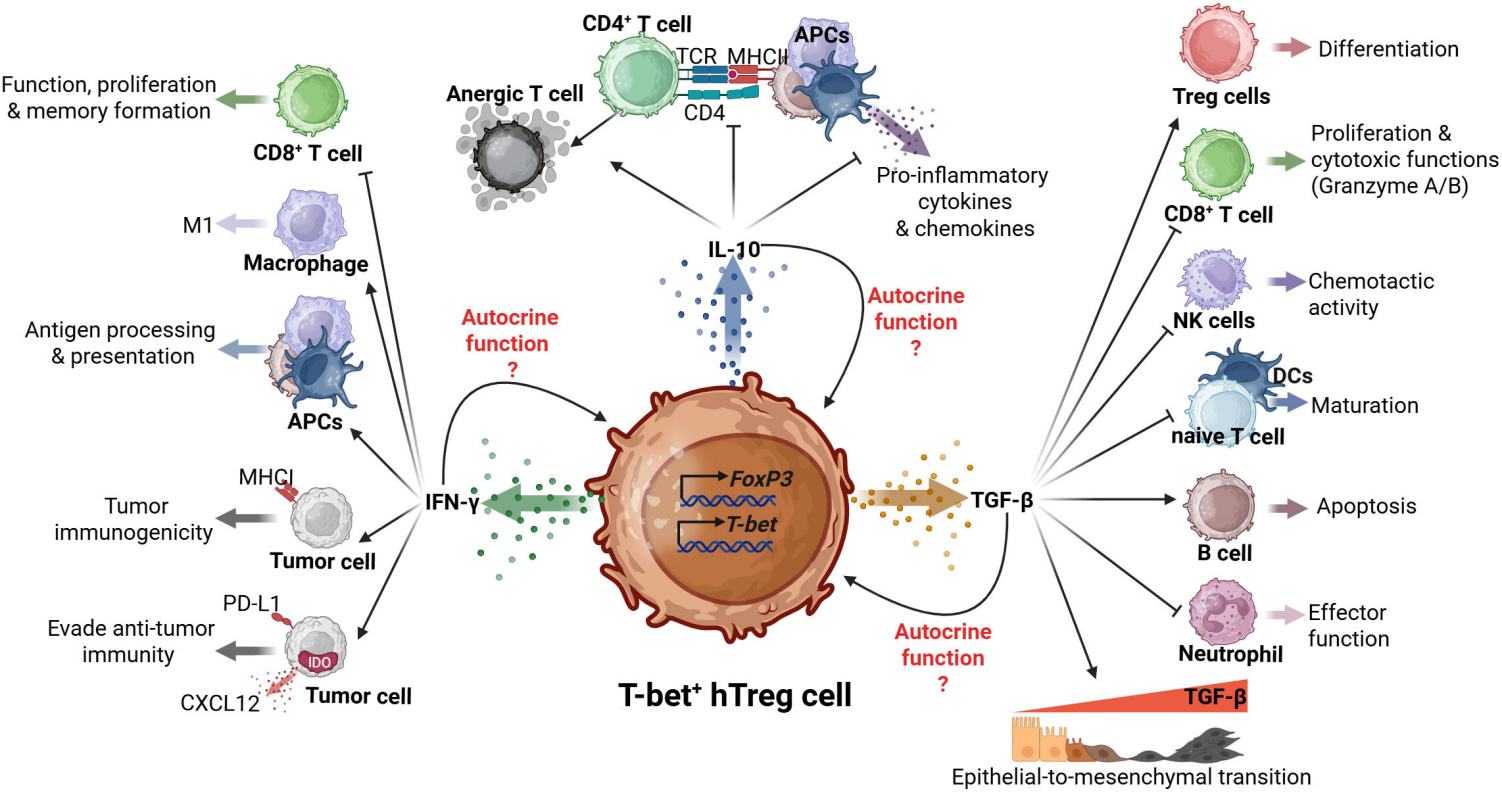
Dual roles of T-bet^+^ hTregs in tumor progression. The T-bet^+^ hTregs, characterized by Foxp3 and T-bet expression, exhibits complex interactions through its mainly produced cytokines (IFN-γ, IL-10, and TGF-β). IL-10 production suppresses CD4^+^ T cell responses and pro-inflammatory cytokine production; potential autocrine function(s) are unclear. TGF-β signaling orchestrates multiple immunomodulatory effects such as Treg differentiation, CD8^+^ T cell regulation, NK cell chemotaxis, and dendritic cell maturation. These functions demonstrate a potential role of T-bet^+^ hTregs in coordinating both immunosuppressive and pro-tumoral responses within the TME. Finally, the IFN-γ production influences antigen presentation through APCs, macrophage polarization, and tumor cell responses, including immunogenicity and immune evasion mechanisms.

Although, T-bet^+^ hTregs produce cytokines such as IFN-γ typically associated with Th1 cell responses, they also secrete IL-10, and transforming growth factor-beta (TGF-β), produced by conventional Treg cells (**Figure 1**) (18, 21).

#### 3.1.1 IL-10

IL-10 is a member of the IL-10 cytokine family with a crucial role in maintaining epithelial tissue integrity, defending against pathogens, and preserving self-tolerance (46). It plays a dual role in cancer immunity exhibiting both antitumor and pro-tumor functions (47). Traditionally, IL-10 was regarded as an immunosuppressive cytokine in the TME. However, recent studies have revealed its antitumor functions. The antitumor or pro-tumor role of IL-10 is linked to the phosphorylation of signal transducer and activator of transcription (STAT) 1 or STAT3. The IL-10 receptor is a heterotetrametric complex consisting of two IL-10Rα and two IL-10Rβ subunits. Upon IL-10 binding, this receptor forms a hexamer, initiating a signaling cascade primarily involving Janus kinase 1 (JAK1) and tyrosine kinase 2 (TYK2), which leads to the phosphorylation of STAT3. Activated STAT3 suppresses excessive immune responses by inhibiting mitogen-activated protein kinase (MAPK), nuclear factor kappa B (NF-κB), and IL-1R production. Conversely, the hexamer can phosphorylate STAT1, enhancing granzyme and IFN-γ production specifically in tumor-resident CD8^+^ T cells. This effect is not observed in CD8^+^ T cells isolated from lymph nodes or in CD4^+^ T cells (48). Therefore, the balance between STAT1 and STAT3 abundance within IL-10R-expressing cells, such as macrophages, dictates the functional outcome of IL-10 signaling. Secretion of IL-10 by T-bet^+^ hTregs requires a strong TCR activation, STAT4 signaling, and IL-12 signaling (49). IL-4 stimulates IL-10 production in CD4^+^ T cells, helping to regulate the balance between pro-inflammatory and anti-inflammatory cytokines while encouraging the development of a T-bet^+^ hTregs phenotype (50). In addition, IL-10 dampens the ability of dendritic cells (DCs) and macrophages to activate antigen-specific CD4^+^ T cells. In activated macrophages, IL-10 signaling downregulates major histocompatibility complex class (MHC) II and CD86, ultimately disrupting the antigen presentation process to CD4^+^ T cells by upregulating the E3 ubiquitin ligase, March1 (51, 52). IL-10 also reduces the expression of MHC-I on tumor cells, resulting in tumor immune escape (53). IL-10 plays a pivotal role in modulating both innate and adaptive immune responses by restricting T cell activation and differentiation in lymph nodes, and suppressing pro-inflammatory activity in tissues. This suppression occurs through the reduction of pro-inflammatory cytokines (e.g., IL-1, IL-6, IL-12, IL-18, and TNF-α) and chemokines (e.g., MCP1, MCP5, RANTES, IL-8, IP-10, and MIP-2), potentially leading to impaired pathogen clearance or reduced immunopathology (54). IL-10 can directly inhibit T cell functions by suppressing proliferation, cytokine production (IFN-γ, IL-2) by CD4^+^ T cells and promoting T cell anergy (54, 55).

#### 3.1.2 Transforming growth factor-beta

TGF-β exhibits a dual role in cancer progression, functioning as a tumor suppressor during the early stages but shifting to promote tumor growth, metastasis, and immune evasion in advanced stages (56–58). TGF-β is produced by a variety of immune and non-immune cells. It is synthesized in an inactive form that requires activation to become functionally active. This activation step serves as a vital regulatory mechanism to control the biological activity of TGF-β (59). Tregs are the primary source of latent TGF-β1 (LTGF-β1) among CD4^+^ T cells, and uniquely express glycoprotein-A repetitions predominant (GARP) on their surface upon activation. GARP functions as a docking receptor for LTGF-β1, facilitating the release of active TGF-β by integrins (60). TGF-β secreted by Tregs functions in both an autocrine and paracrine manner (61). In normal cells and early-stage cancers, TGF-β acts as a tumor suppressor by inhibiting cell proliferation through repression of c-Myc and induction of cyclin-dependent kinase inhibitors (CDKIs) (62). Within the TME, TGF-β can be secreted by tumor cells, fibroblasts, immune cells, and endothelial cells, and promotes epithelial to mesenchymal transition (EMT) in cancer cells. This process allows advanced-stage tumor cells to migrate from their primary site, enter the bloodstream, colonize distant locations, and form secondary tumors. TGF-β also induces Foxp3 expression, and supports differentiation of naive CD4^+^ T cells into peripherally-induced Tregs (pTregs), while simultaneously suppressing their conversion to other immune cell types. It inhibits the proliferation and function of cytotoxic CD8^+^ T cells, reduces the cytotoxicity and chemotactic activity of NK cells, and impairs the maturation of DCs and naïve CD4^+^ T cells (63, 64). TGF-β also impairs the effector functions of neutrophil and induces apoptosis in B cells. In addition to promoting Foxp3 expression in Tregs, TGF-β inhibits IFN-γ production, and blocks expression of multiple NK cell-related makers, including MHC class I chain-related molecule A (MICA), granzyme A/B, natural killer group 2 member D (NKG2D), natural cytotoxicity receptor 3 (NKP30), and perforin, thereby suppressing immune activity (63). In transformed epithelial cells, TGF-β supports metastasis by upregulating IL-11, parathyroid hormone-related peptide (PTHrP), and matrix metalloprotease (MMP) 9. It also enhances the expression of connective tissue growth factor (CTGF), vascular endothelial growth factor (VEGF), and MMP2, to promote angiogenesis (63). Overall, the pro-tumor effects of TGF-β outweigh its tumor-suppressive functions. Although bulk RNA-seq analysis revealed low GARP expression in T-bet^+^ hTregs (25), whether GARP in this subset can activate latent TGF-β and modulate T-bet^+^ hTreg function in an autocrine manner remains to be determined.

#### 3.1.3 IFN-γ

While IFN-γ is widely recognized for its crucial role in stimulating anti-tumor immune responses, it can also have a paradoxical protumor function depending on its concentration and the context within the TME (65). Tregs can acquire Th1-like effector properties while maintaining Foxp3 expression, resulting in IFN-γ^+^ T-bet^+^ hTregs. This process is stimulated by IFN-γ, IL-12, or IL-27 and ultimately driven by activation of the PI(3)K-Akt-FOXO signaling pathway (66). Culturing thymic Treg (tTreg) cells with IFN-γ and TGF-β upregulates T-bet and CXCR3 expression. Notably, in the absence of IFN-γ, TGF-β downregulates T-bet expression through a Foxp3-independent mechanism. T-bet also directly enhances the expression of *Ifng* and *IL12rb* by binding to their loci. IFN-γ plays a key role in the differentiation of T-bet^+^ hTregs and is also produced by these cells (20). IFN-γ can activate macrophages and promotes their differentiation into M1 macrophages. IFN-γ also enhances tumor immunogenicity by increasing MHC class I expression on tumor cells, improving antigen presentation, and activating cytotoxic CD8^+^ T cells to target and eliminate the tumor (67). Deficiency in IFN-γ may cause spontaneous tumor development (60); but in some settings it may function as a pro-tumor molecule. It was reported that IFN-γ can induce programmed cell death ligand 1 (PD-L1), indoleamine 2, 3 dioxygenase (IDO), and CXCL12 in tumor cells supporting their antitumor immune evasion (68–70). Of note, the net pro-tumor or antitumor effects of IFN-γ like that of many other cytokines is determined by a broader network of factors, including the tumor microenvironment (e.g., cytokine milieu, immune cell composition, hypoxia, and metabolic state), tumor type, and the timing and duration of IFN-γ signaling (acute versus chronic exposure) (65, 71, 72). For instance, IFN-γ in melanoma acts through a negative feedback mechanism to constrain anti-tumor immune responses by diminishing the longevity of stem-like T cells (73). During acute and chronic infections, T-bet^+^ hTregs suppress the function, proliferation, and memory formation of CD8^+^ T cells (74). A study using a USP15-deficient mouse model revealed that elevated IFN-γ secretion by T cells transforms the TME into an immunosuppressive state, promoting the accumulation of T-bet^+^ hTregs and CD11b^+^Gr-1^+^ myeloid-derived suppressor cells at the tumor site (70). Despite the well-characterized role of IFN-γ in shaping the TME, the mechanistic function of IFN-γ secreted specifically by T-bet^+^ hTregs remains poorly understood. It is unclear whether the IFN-γ produced by these cells dictates an immunosuppressive TME. Furthermore, the potential autocrine effects of IFN-γ on T-bet^+^ hTregs is yet to explored. This raises intriguing questions about whether IFN-γ regulates the stability, suppressive function, or metabolic activity of T-bet^+^ hTregs through self-signaling mechanisms. Further investigation is needed to clarify these pathways and their implications in tumor immunity and therapeutic interventions.

### 3.2 Expression of immune checkpoint

#### 3.2.1 Programmed cell death 1

**Figure 2:**
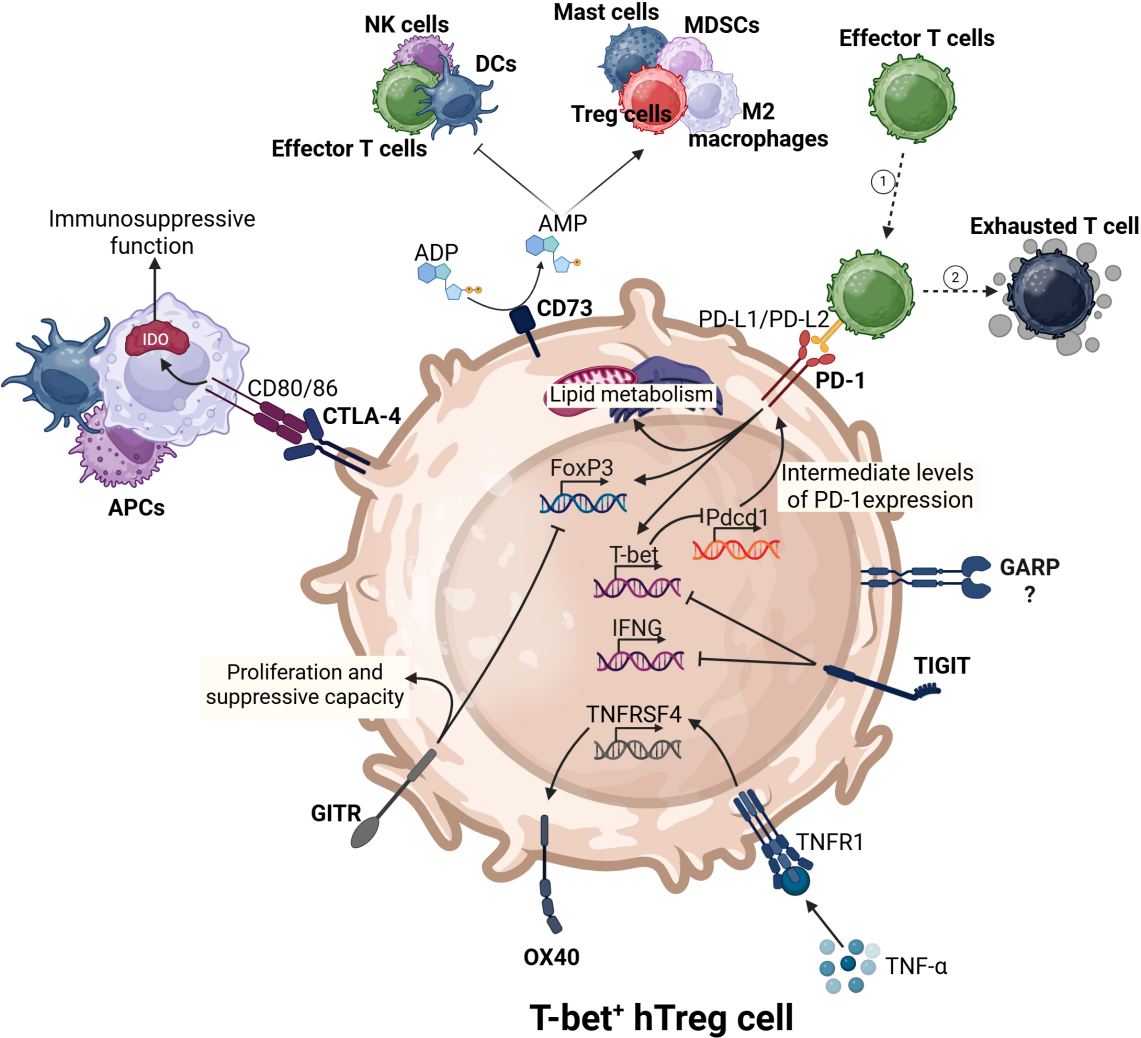
The complex immunoregulatory interactions of T-bet^+^ hTregs. T-bet^+^ hTregs express key transcription factors (Foxp3 and T-bet). These cells utilize various immune checkpoints to regulate their activities on the surrounding immune environment. For instance, CD73 promotes pro-tumor immune responses and suppresses antitumor immunity. PD-1 induces the expression of both Foxp3, T-bet, and lipid metabolism. CTLA-4, through the upregulation of IDO in antigen-presenting cells (APCs) can enhance the immunosuppressive nature of the TME.

PD-1 is a immunosuppressive checkpoint expressed by various immune cells, including macrophages, B lymphocytes, DCs, tumor-specific activated T cells, and NK cells under conditions of chronic antigen exposure (75). In addition to activated T cells, tumor infiltrated CD8^+^ T cells and Tregs also express PD-1, which interacts with its ligands, PD-L1 and PD-L2 (76, 77). It was demonstrated previously that activated T-bet^+^ hTregs express high levels of PD-1 (14). TCR signaling triggers the activation of various transcription factors, including NFAT2, AP-1, Notch, FOXO1, and TOX, leading to the upregulation of PD-1 (77). PD-1 is critical for the extrathymic differentiation of pTreg cells in the periphery. PD-1-deficient conventional CD4^+^ T cells show a markedly reduced ability to differentiate into pTregs in various *in vivo* settings (76). PD-1 signaling also promotes lipid metabolism in tumor-infiltrating Tregs, contributing to their proliferation and suppressive function (**Figure 2**) (78). Moreover, PD-1 signaling enhances Foxp3 expression in Tregs and strengthens their suppressive characteristics (79). Through PD-1 and PD-L1/PD-L2 signaling with APCs, Tregs maintain immune tolerance in CD4^+^ and CD8^+^ T cells. In the TME, Tregs can promote upregulation of PD-1 in tumor-infiltrating antitumor T cells, leading to their exhaustion, and consequently promoting immune evasion tumor cells (80). It was proposed that intermediate levels of PD-1 expression promote T-bet expression during chronic infection, and support the survival of T-bet^+^ cells. In turn, T-bet maintains PD-1 expression at intermediate levels by repressing *Pdcd1* transcription (81). While PD-1 is known to play a significant role in immune regulation, the specific mechanisms by which PD-1 on T-bet^+^ hTregs may modulate immune responses against tumors remain unclear and warrant further investigation.

#### 3.2.2 Cytotoxic T-lymphocyte associated protein 4

CTLA-4 is an inhibitory receptor on T cells and a member of the CD28 family. CD80 and CD86 are known ligands of CTLA-4, and are mostly present on the surface of APCs. Of note, CD80 and CD86 binding to CTLA-4 inhibits T cell activation, while binding to CD28 to activates T cells. Therefore, CTLA-4 and CD28 compete for binding to CD80 or CD86 (82). CTLA-4 is crucial for the suppressive function of Tregs. It interacts with CD80 and CD86 on dendritic cells, transmitting inhibitory signals that downregulate CD80 and CD86 expression and upregulate IDO in dendritic cells, promoting immune tolerance (83, 84). Under normal physiological conditions, IDO maintains immune tolerance; however, in the TME, it acts as an immunosuppressive enzyme (85). Tregs express higher levels of CTLA-4 compared to conventional T cells, and have a greater affinity for binding CD80 and CD86 than CD28. As a result, Tregs outcompete with conventional T cells for CD80 and CD86 binding, leading to reduced activation of conventional T cells (86). Despite reports that T-bet^+^ hTregs express CTLA-4, the mechanism by which CTLA-4 regulates their suppressive function remains unclear (14).

#### 3.2.3 Glucocorticoid-induced TNFR family related protein

As a member of the TNF receptor family, the GITR immune checkpoint is expressed at low levels on CD8^+^ and CD4^+^ T cells but highly expressed on Tregs (87, 88). Its expression levels are frequently associated with proliferation and the immunosuppressive activity of Tregs (89, 90). GITR expression has been reported on T-bet^+^ hTregs (91). Anti-GITR treatment shifts the immunosuppressive TME of glioblastoma to an immunostimulatory one by specifically targeting glioblastoma Tregs. This treatment converts immunosuppressive Tregs into antitumor Th1-like CD4^+^ T cells, enhancing the immune response and overcoming resistance to α-PD1 therapy in experimental glioblastoma models (92). The function of GITR in TME is somewhat controversial due to its context-dependent role in T cells. Some studies have reported that triggering GITR signaling with Fc-GITR-L inhibits intratumoral Treg suppressive function but drives CD4^+^ effector T cell to proliferate (87). Overall, the role of GITR in Tregs, particularly in T-bet^+^ hTregs remains an area that requires further investigation. Understanding how GITR signaling influences the stability, suppressive function, and interaction of T-bet^+^ hTregs within the TME could provide valuable insights into their contribution to immune regulation in cancer. Due to the complex nature of Treg cell plasticity, investigating the mechanism behind the function of GITR could help refine therapeutic strategies that target Tregs to enhance antitumor immunity.

#### 3.2.4 Tumor necrosis factor receptor superfamily, member 4

OX40 is a member of the tumor necrosis factor (TNF) receptor/nerve growth factor (NGF) receptor superfamily and a costimulatory molecule expressed on T cells, including Tregs. It plays a complex role in regulating Treg function, typically acting as a negative regulator. Excessive OX40 signaling can impair the suppressive abilities of Tregs and enhance effector T cell responses, potentially disrupting immune tolerance (93, 94). TNF-α signaling elevates OX40 expression and enhances the suppressive capacity of Tregs (19). OX40^−^ T-bet^+^ hTregs express IFN-γ and exhibit reduced suppressive function, whereas OX40^+^ T-bet^+^ hTregs have a suppressive effect on immune responses. OX40^+^ Tregs are more abundant in cirrhosis and TME, while OX40^−^ Tregs preferentially accumulate in non-cirrhotic chronic HCV liver tissue. OX40 stimulation has been explored as a potential strategy to abolish Treg function and target tumors (19). It was reported that Tregs express higher levels of OX40 in comparison to CD8^+^ T cells and blockade of OX40 reduced Tregs (95). However, the precise mechanisms underlying the differential effects of OX40 signaling on T-bet^+^ hTregs remain poorly understood, particularly in terms of their functional plasticity and role in the TME.

#### 3.2.5 T cell immunoreceptor with Ig and ITIM domains

TIGIT is a key inhibitory receptor on Tregs that suppresses pro-inflammatory Th1 and Th17 responses, thereby maintaining immune tolerance (96). The expression of TIGIT has been reported on T-bet^+^ hTregs (24); however, despite the well-established role of TIGIT in various T cell subsets, its specific function within T-bet^+^ hTregs remains poorly characterized and is supported by only limited experimental evidence. TIGIT signaling inhibits the production of IFN-γ and the expression of T-bet, while also restoring the suppressive function of Tregs treated with IL-12. Blocking FOXO1 function eliminates the protective effects of TIGIT, suggesting that TIGIT signaling enhances the nuclear localization of FOXO1 (97).

#### 3.2.6 CD73

T-bet^+^ hTregs express higher levels of CD73 compared to other hybrid Tregs subsets (18). Adenosine triphosphate (ATP) and adenosine diphosphate (ADP) can be released to the extracellular space due to inflammatory conditions such as hypoxia, acute injury, and cancer. Extracellular ATP is dephosphorylated to ADP by CD39, and ADP is further dephosphorylated to adenosine monophosphate (AMP) by CD73 (98). Extracellular AMP triggers intracellular signaling through adenosine receptors such as 1R, A2AR, A2BR, and A3R. These signaling activities result in an anti-inflammatory responses, including boosting accumulation of Tregs, myeloid-derived suppressor cells (MDSCs) and M2 macrophages in tumors (98, 99). CD73 expression has been reported in a wide range of tumors, including ovarian cancer, melanoma, and prostate cancer (98). T-bet^+^ hTregs might use a similar strategy to suppress antitumor immunity; however, the exact mechanism of CD73 function in these cells remains to be discovered.

### 3.3 Chemokine receptors

#### 3.3.1 C-X-C motif chemokine receptor 3

As a chemokine receptor, CXCR3 plays a crucial role in T cell trafficking and function. CXCR3 is highly expressed on Th1-type CD4^+^ T cells and effector CD8^+^ T cells, and its signaling can be triggered by CXCL9, CXCL10, and CXCL11 (100). T-bet upregulates CXCR3 to enhance the migration of Th1 effector cells to inflammatory sites. Similarly, Foxp3^+^ Tregs can also express T-bet in response to IFN-γ, which drives CXCR3 expression and facilitates the recruitment of these suppressive T cells to inflammatory sites (101). In contrast to Tregs, T-bet^+^ hTregs express high levels of CXCR3 (14). Within the TME, different cells in response to IFN-γ (e.g. monocytes, endothelial cells, fibroblasts, and cancer cells) secrete CXCL9, CXCL10, and CXCL11 (102). Therefore, paracrine actions of CXCL9, CXCL10, and CXCL11 attract immune cells including Tregs to the TME. CXCR3 also plays an important role in the accumulation and immune suppressive function of tumor-infiltrating Tregs. Treg expression of CXCR3 enables their interaction with type I dendritic cells within the TME to limit the antitumor activities of CD8^+^ T cells (103). T-bet^+^ hTregs influence CD8^+^ T cell activation in the tumor-draining lymph node, although this effect appears to be unrelated to CXCR3 activity (104). CXCR3^+^ Treg cells demonstrated a greater ability to suppress CD4^+^CD44^+^ Th1 effector cells obtained from LCMV-infected mice *in vitro* (105).

#### 3.3.2 C-C chemokine receptor type 5

CCR5 (CD195) plays an important role during T cell migration by guiding activated T cells to sites of inflammation (106). CCR5 expression is markedly higher on Th1-like Tregs, making it a reliable marker for identifying T-bet^+^ hTregs (91). T-bet^+^ hTregs in the peripheral blood of untreated, relapsing-remitting MS (RRMS) patients exhibit higher levels of T-bet, CXCR3, CCR5, and IFN-γ, along with reduced levels of TGF-β and CTLA-4 (19). CCR5 expression on T-bet^+^ hTregs allows their migration to inflammatory sites where CCL5, the ligand for CCR5 is produced, and potentially facilitates their immune suppressive function (107).

### 3.4 Self-regulation and plasticity

Cell fate is determined by the interplay between extrinsic environmental signals and intrinsic, cell-autonomous programs. The relative influence of these factors varies by cell type and developmental stage (108). *Self-regulation:* While Th1 responses clear pathogens, it can also damage healthy host tissues (109). CD4^+^ T cells including Th1 cells can utilize IL-10 as a self-regulatory mechanism during pathogen clearance to reduce immunopathology (110). During Leishmania major, Toxoplasma gondii infection and excessive inflammation, IL-10 production by Th1 cells is suggested to function as a self-regulatory mechanism that helps limit tissue damage (109, 111). Indeed, therapeutically inducing the switch from IFN-γ^+^ to IL-10^+^ Th1 cells is a promising approach; however, this depends on identifying the molecular checkpoints that govern Th1 development and evaluating their therapeutic potential (112). Tregs also possess an intrinsic self-regulatory system. Foxp3, in particular, interacts with multiple cofactors to create complex positive and negative feedback loops that precisely control Treg development, stability, and suppressive function (113). For instance, the positive feedback loop is reinforced by IL-2, which binds to the high-affinity IL-2 receptor on most Treg cells, preventing apoptosis through upregulation of MCL1 expression (114). *Plasticity:* While IL-4 treatment induces Th1 cells to upregulate GATA-3 and adopt a hybrid T-bet^+^GATA-3^+^ Th1/Th2 phenotype, high T-bet expression remains essential for maintaining Th1 phenotypic stability (115). In tumors, IFN-γ producing Th1 cells can differentiate into Treg cells under the influence of TGF-β signaling (116). Plasticity in Tregs is controlled by both metabolic and transcriptional mechanisms. T-bet^+^ hTregs differentiate via IL-12–driven activation of the PI3K/Akt/Foxo pathway, with HIF-1α promoting glycolysis and directly binding the Ifng gene to enhance IFN-γ expression (117). T-bet^+^ hTregs can arise from Th1 cells or conventional Tregs through cellular plasticity (116, 118, 119). However, the extent to which self-regulation and plasticity contribute to their development from each source remains unclear. Overall, understanding the balance between self-regulation and plasticity during differentiation of T-bet^+^ hTregs is essential to unveiling how they establish and maintain their identity.

## 4 T-bet^+^ hTregs as a new candidate to fight cancer

Tregs play a crucial role in maintaining immune homeostasis by suppressing excessive immune responses. Nevertheless, within the TME Tregs can inhibit antitumor immunity, promoting tumor progression. In cancer patients, increased infiltration of Tregs within tumor tissues is often associated with poor clinical outcomes (120). As a result, targeting Tregs has become a key focus in cancer immunotherapy. Depletion of Tregs has been shown to significantly boost antitumor immunity; however, systemic depletion of Tregs can lead to severe autoimmune diseases (7). Different immunotherapies have been examined to target Tregs, including anti-CTLA-4 agents such as ipilimumab and tremelimumab, but their effectiveness in depleting intratumoral Tregs in humans has been limited. It was previously shown that while these agents increase infiltration of CD4^+^ and CD8^+^ T cells within tumors, they do not significantly reduce the number of Foxp3^+^ Tregs in the tumor microenvironment (121). Immune checkpoint blockade therapy often produces longer-lasting responses compared to chemotherapy or targeted treatments (122, 123). Yet, as global clinical data continues to grow, its limitations and adverse effects are becoming evident. A significant challenge with immune checkpoint blockade therapy is its low efficacy in many cancers, with response rates typically ranging from 10% to 30%. Moreover, for some major cancer types, such as microsatellite-stable colorectal cancer, anti-PD-1/PD-L1 therapy demonstrates minimal effectiveness (124). In addition to immune checkpoint blockade therapies, other strategies have been employed to target Tregs in cancer, including antibody-drug-conjugates (ADC), small-interfering RNA (siRNA), and peptide-based approaches. Various clinical trials and preclinical studies have used these strategies to target molecules such as CD25, CCR4, CTLA-4, STAT3, PD-1, Foxp3, and 4-1BB (CD137) in the TME. More specifically, peptide targeting has been used to target Foxp3, β-catenin, TGF-β1, NRP-1, and CXCR4 in Tregs within tumors. Although knowledge about these strategies is growing, their side effects remain largely unknown (125). Therefore, more effective therapies are still required to combat cancer. A promising strategy involves appreciating the heterogeneity of Tregs within the TME and investigating the roles of specific Tregs subsets. This approach could open new opportunities for developing treatments that selectively target these subsets in cancer patients.

Tregs within tumors exhibited elevated expression of T-bet, which was crucial for their activity in the TME. Furthermore, CD39 was prominently expressed on T-bet^+^ Tregs in both mouse and human tumors and played a key role in enabling these cells to suppress CD8^+^ T cell responses (116). For instance, the presence of T-bet^+^ hTregs has been reported in various human cancers, including ovarian cancer, lung cancer, colorectal cancer, hepatocellular carcinoma, and oral squamous cell carcinoma (20). This Treg subset is stable even under inflammatory or otherwise unfavorable conditions (126). T-bet^+^ hTregs represent a specific subset of Tregs characterized by the expression of CXCR3. Therefore, combining immunotherapies such as anti-CTLA-4 and anti-PD-1 with CXCR3 blockade could offer a novel approach to targeting T-bet^+^ hTregs. Several CXCR3 blockers have been reported in preclinical studies for various diseases, including autoimmune, inflammatory diseases, and transplant rejection (127). Preclinical studies using AMG487, CXCR3 blocker, demonstrated significant reductions in metastasis in murine models of breast cancer and osteosarcoma (128, 129). AMG487 has also been evaluated in clinical trials for the treatment of psoriasis and rheumatoid arthritis (130). In another clinical trial, ACT-777991 was evaluated for safety, tolerability, and pharmacokinetics in single- and multiple-ascending doses in healthy subjects. However, neither AMG487 nor ACT-777991 has yet been assessed in clinical trials for cancer (131). Inhibition of CXCR3 on T-bet^+^ hTregs could hinder their migration to tumor sites, potentially reducing their ability to suppress local immune responses. However, targeting CXCR3 may also impair the migration of antitumor immune cells to the tumor site. By selectively inhibiting the recruitment of T-bet^+^ hTregs to the TME, it may be possible to enhance the effectiveness of existing therapies and improve clinical outcomes. Furthermore, targeting this subset could help mitigate the off-target effects seen with broader Treg depletion strategies, minimizing the risk of autoimmune complications. Thus, refining our understanding of Treg subtypes and their specific roles in cancer immunity is crucial for advancing therapeutic strategies aimed at overcoming the challenges associated with current cancer immunotherapies.

## 5 Discussion and future perspectives

In conclusion, the discovery of T-bet^+^ hTregs has added a new layer of complexity to our understanding of tumor immunology and the potential for cancer immunotherapy. These cells, characterized by the co-expression of Foxp3 and T-bet, along with the production of IFN-γ, represent a unique subset of Tregs with high plasticity and context-dependent functions within the TME. T-bet^+^ hTregs exhibit both pro-tumor and antitumor properties, highlighting their adaptability to the TME. They suppress antitumor immunity through various mechanisms, including cytokine production (IL-10, TGF-β, IFN-γ, and TNF-α) and expression of immune checkpoint molecules (PD-1, CTLA-4, GITR, OX40, and TIGIT). Their high expression of chemokine receptors, particularly CXCR3 and CCR5, facilitates their migration to inflammatory sites and tumors, potentially enhancing their immunosuppressive functions. The heterogeneity of Tregs within the TME, including the T-bet^+^ hTregs subset, presents both challenges and opportunities for cancer immunotherapy. While global Treg depletion has shown promise in boosting antitumor immunity, it carries the risk of severe autoimmune complications. Targeting specific Tregs subsets, such as T-bet^+^ hTregs could offer a more nuanced approach to cancer treatment. However, strategies to selectively target specific cell types remain limited. For example, the emerging PROTAC (Proteolysis-Targeting Chimera) platform can efficiently degrade a defined protein within a cell, yet achieving selective degradation of a specific protein in a particular cell type introduces additional layers of complexity and challenges in feasibility. If such a system were available, selectively targeting both FOXP3 and T-bet, rather than each factor individually, in T-bet^+^ hTreg cells could more effectively diminish their capacity to suppress antitumor immunity. Currently, feasible strategies for targeting T-bet^+^ hTregs include combining existing immunotherapies (e.g., anti-CTLA-4 and anti-PD-1) with CXCR3 blockade or developing drugs that specifically inhibit CXCR3 expression on these cells. Such approaches could prevent their migration to tumor sites and reduce their immunosuppressive effects, potentially enhancing the efficacy of current therapies while minimizing off-target effects. In addition, emerging technologies, including spatial transcriptomics, hTreg-specific gene editing models, and single-cell multi-omics approaches (such as scRNA-seq and CITE-seq), now enable precise dissection of T-bet^+^ hTreg heterogeneity, localization, and function within the TME. These tools provide unprecedented insight into their suppressive mechanisms and interactions with other immune cells. Together, they pave the way for developing targeted strategies to modulate Treg subsets while minimizing off-target effects. It is worth noting that only limited information is currently available regarding the specific roles of T-bet^+^ hTregs in cancer and their potential as therapeutic targets. More research is needed to fill these knowledge gaps and inform the development of more effective cancer immunotherapies.
